# Supplementary material for: A cell cycle-dependent CRISPR-Cas9 activation system based on an anti-CRISPR protein shows improved genome editing accuracy
Source: Commun Biol. 2020 Oct 23;3:601. doi: 10.1038/s42003-020-01340-2 (PMC7584632; doi:10.1038/s42003-020-01340-2)
Supplement: Supplementary file 1 — Supplementary Information [file 42003_2020_1340_MOESM1_ESM.pdf]

Supplementary Information

A Cell Cycle-dependent CRISPR-Cas9 Activation System Based on an Anti-CRISPR Protein Shows Improved Genome Editing Accuracy

Daisuke Matsumoto, Hirokazu Tamamura, and Wataru Nomura*

1Institute of Biomaterials and Bioengineering, Tokyo Medical and Dental University, 2-3-10 Kandasurugadai, Chiyoda-ku, Tokyo, 101-0062, Japan

2Graduate School of Biomedical and Health Sciences, Hiroshima University, 1-2-3 Kasumi Minami-ku, Hiroshima, 734-8553, Japan

** To whom correspondence should be addressed. Tel: +81-82-257-5308; Fax: +81-82-257-5309; Email: wnomura@hiroshima-u.ac.jp*

†Present Address: Daisuke Matsumoto, Department of Chemistry, The Scripps Research Institute, La Jolla, CA, 92037, USA

Supplementary Information: Figures

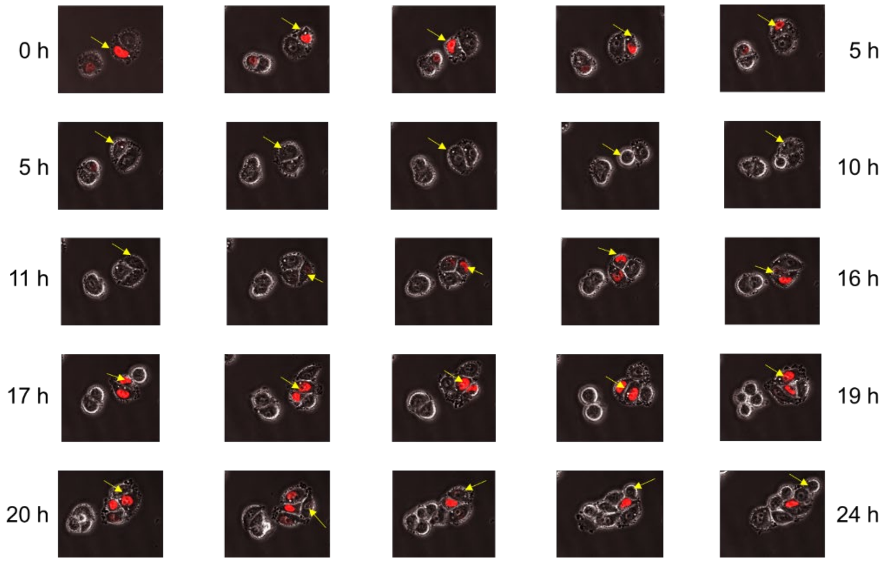

Supplementary Figure 1. Change of the expression level of mKO2-Cdt1 (Fucci) depending on time-lapse. Time indicate hours from the start of observation.

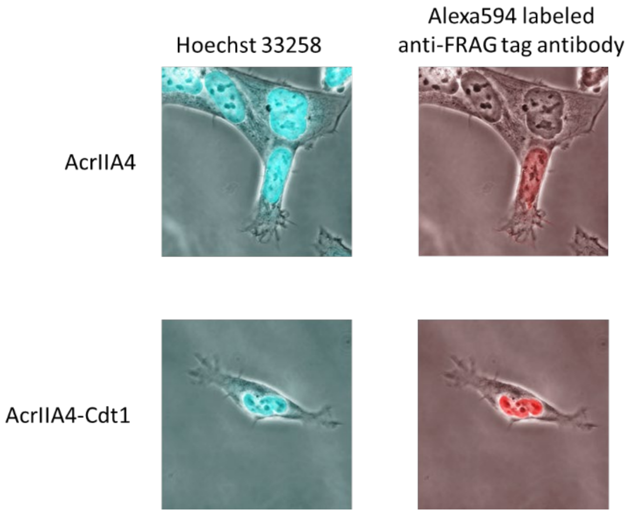

Supplementary Figure 2. Confirmation of expression and localization of AcrIIA4 and AcrIIA4-Cdt1in 293A cell. Immunostaining was performed 48 h after transfection. AcrIIA4 and AcrIIA4-Cdt1 were labeled by Alexa594 labeled anti-FLAG tag antibody. Nucleus was stained by Hoechst 33258.

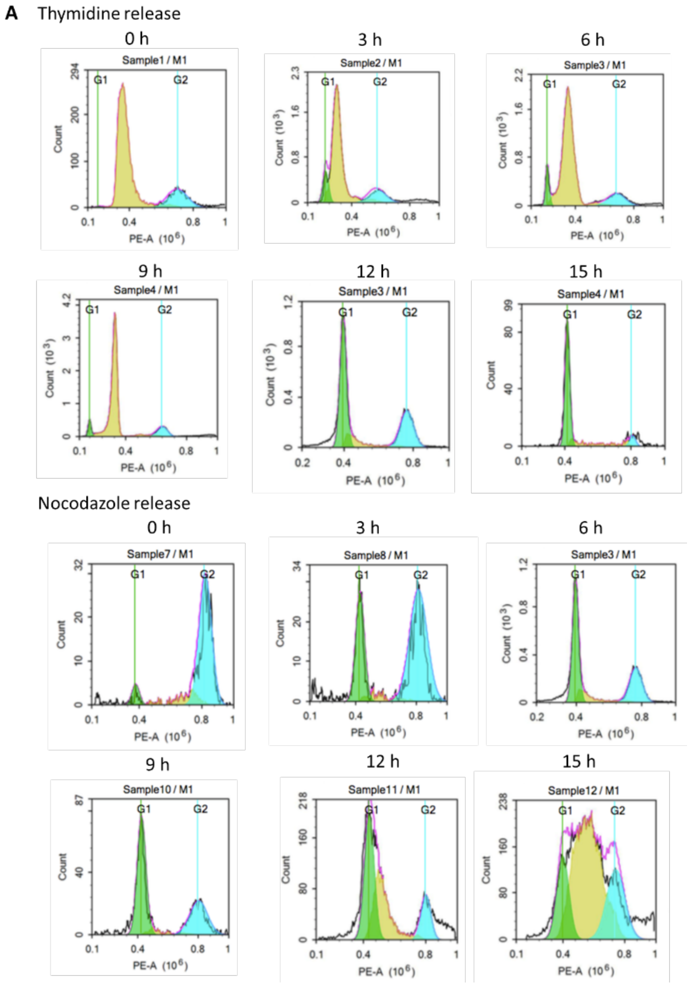

Supplementary Figure 3. FACS analysis for cell cycle. Cells were synchronized by double Thymidine block or Nocodazole. Cell cycle population was analyzed at each time after release from the drugs. Green range shows G_1 phase. Yellow range shows S phase. Blue range shows G_2 phase.

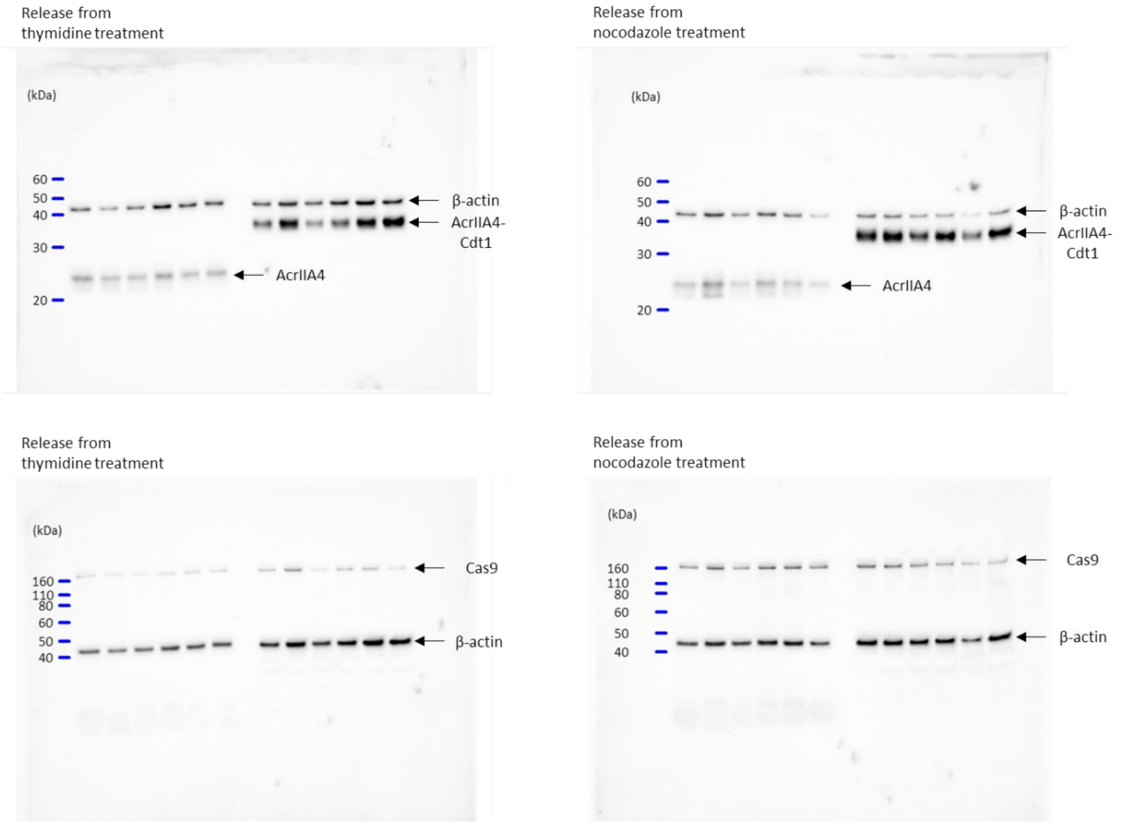

Supplementary Figure 4. Western blotting images for Fig. 3. Molecular sizes are shown based on the protein marker images. Cells were synchronized by double Thymidine block or Nocodazole.

H2B target site

TCAGACACAAGACTTCAACTCTTCACCTTATTTGCATAAGCGATTCTATATAAAAGCGCCTTGTCATACCCTGCTCAC

GCTGTTTTTCCTTTTCGTTGGCGCTTTATAGCTACACAGTGCTATGCCAGAGCCAGCGAAGTCTGCTCCCGCCCCGAA

AAAGGGCTCCAAGAAGGCGGTGAC

Sequence after HDR

TCAGACACAAGACTTCAACTCTTCACCTTATTTGCATAAGCGATTCTATATAAAAGCGCCTTGTCATACCCTGCTCAC

GCTGTTTTTCCTTTTGGATCCCTCGAGGCGGCCGCCGTTGGCGCTTTATAGCTACACAGTGCTATGCCAGAGCCAGCG

AAGTCTGCTCCCGCCCCGAAAAAGGGCTCCAAGAAGGCGGTGAC

AAVS1 target site

AGGAGGAGGCCTAAGGATGGGGCTTTTCTGTCACCAATCCTGTCCCTAGTGGCCCCACTGTGGGGTGGAGGGGACAGA

TAAAAGTACCCAGAACCAGAGCCACATTAACCGGCCCTGGGAATATAAGGTG

Sequence after HDR

AGGAGGAGGCCTAAGGATGGGGCTTTTCTGTCACCAATCCTGTCCCTAGTGGCCCCACTGTGGAAGCTTGGATCCGGA

GGGGACAGATAAAAGTACCCAGAACCAGAGCCACATTAACCGGCCCTGGGAATATAAGGTG

EMX1 target site

CAGGTGAAGGTGTGGTTCCAGAACCGGAGGACAAAGTACAAACGGCAGAAGCTGGAGGAGGAAGGGCCTGAGTCCGAG

CAGAAGAAGAAGGGCTCCCATCACATCAACCGGTGGCGCATTGCCACGAAGCAGGCCAATGGGGAGGACATCGATG

Sequence after HDR

CAGGTGAAGGTGTGGTTCCAGAACCGGAGGACAAAGTACAAACGGCAGAAGCTGGAGGAGGAAGGGCCTGAGTCCGAG

CAGAAGAAGCTTGGATCCAAGGGCTCCCATCACATCAACCGGTGGCGCATTGCCACGAAGCAGGCCAATGGGGAGGAC

ATCGATG

VEGFA target site

GAAGCTGGGTGAATGGAGCGAGCAGCGTCTTCGAGAGTGAGGACGTGTGTGTCTGTGTGGGTGAGTGAGTGTGTGCGT

GTGGGGTTGAGGGCGTTGGAGCGGGGAGAAGGCCAGGGGTCACTCCAGGATTCCAATAGATCTGTGTGTCCCTCTC

Sequence after HDR

GAAGCTGGGTGAATGGAGCGAGCAGCGTCTTCGAGAGTGAGGACGTGTGTGTCTGTGTGGGTGAGTGAGTGTGTAAGC

TTGGATCCTGTGGGGTTGAGGGCGTTGGAGCGGGGAGAAGGCCAGGGGTCACTCCAGGATTCCAATAGATCTGTGTGT

CCCTCTC

Supplementary Figure 5. Sequences around target sites and change of the sequence by HDR. Yellow highlights show seed sequences for SpyCas9 recognition and underlines indicate PAM sequences. Red characters are inserted sequences by HDR.

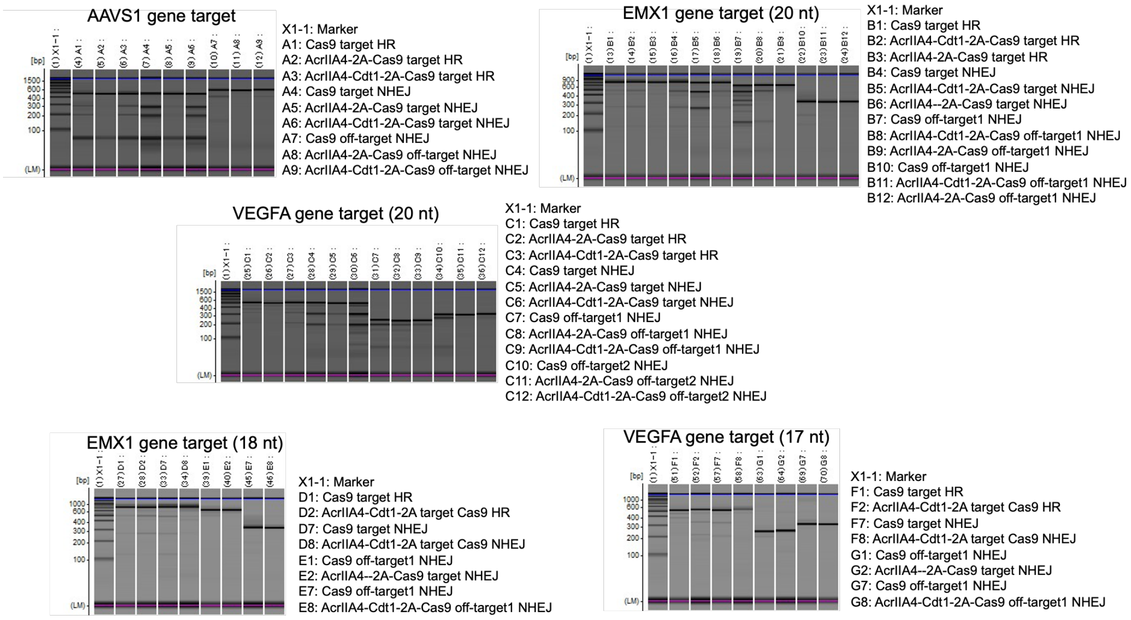

Supplementary Figure 6. Images of each result in Figs. 6 and 7. These images were made from the peak intensities by MultiNA viewer software.

Supplementary Table 1

Name	Sequence (5' to 3')
CMV Forward	CGCAAATGGGCGGTAGGCGTG
BamHI_NLS-AcrIIA4_Fw	ctcctggatccgccaccatggGTGGCCCGAAGAAGAAACGGAAGGTAGGTGGCggcaatataaatgacctcataag
Acr-REsite_XbaI_Rv	GGAGGATCTAGATTACTCGAGCGGCCGCCAGTGT
pEBMultiHyg long Fw	ccgggggatccactagttctagagcGCCACCATGGGTGGCCCG
2A-Cas9	gacttcctctgccctcCTCGAGCGGCCGCCAGTG
NLS-Acr long Fw	GcggccgctcgaggagggcagaggaagtctgctaacatgcggtgacgtcgaggagaatcctggcccaGACAAGAAGTACTCCATTGGGC
pEBMulti-Hyg long Rv	atggctgattatgatctagagtcgcTCACACCTTCCTCTTCTTCTTGG
NLS-Acr-Cdt1 long Fw	CcaggacaccatcgagggcagaggaagtctgctaacatgcggtgacgtcgaggagaatcctggcccaGACAAGAAGTACTCCATTGGGC
pEBCas9 Fw	ccgggggatccactagttctagagcGCCACCATGGACAAGAAGTAC
pEBCas9 Rv	atggctgattatgatctagagtcgcTCACACCTTCCTCTTCTTCTTGG
gRNA_H2B_Fw	TTTCTTGGCTTTATATATCTTGTGGAAAGGACGAAACACCgctataaagcgccaacgaaa
gRNA_H2B_Rv	GACTAGCCTTATTTTAACTTGCTATTTCTAGCTCTAAAACTTTCGTTGGCGCTTTATAGC
gRNA_AAVS1_Fw	TTTCTTGGCTTTATATATCTTGTGGAAAGGACGAAACACCGTGGCCCCACTGTGGGGTGG
gRNA_AAVS1_Rv	GACTAGCCTTATTTTAACTTGCTATTTCTAGCTCTAAAACCCACCCCACAGTGGGGCCAC
gRNA_EMX1_Fw	TTTCTTGGCTTTATATATCTTGTGGAAAGGACGAAACACCGAGTCCGAGCAGAAGAAGAA
gRNA_EMX1_Rv	GACTAGCCTTATTTTAACTTGCTATTTCTAGCTCTAAAACTTCTTCTTCTGCTCGGACTC
gRNA_VEGFA_Fw	TTTCTTGGCTTTATATATCTTGTGGAAAGGACGAAACACCGGTGAGTGAGTGTGTGCGTG
gRNA_VEGFA_Rv	GACTAGCCTTATTTTAACTTGCTATTTCTAGCTCTAAAACCACGCACACACTCACTCACC
ssODN_AAVS1	TATATTCCCAGGGCCGGTTAATGTGGCTCTGGTTCTGGGTACTTTTATCTGTCCCCTCCGGATCCAAGCTTCCACAGTGGGGCCACTAGGGACAGGATTGGTGACAGAAAAGCCCCATCCTTAGGCCTCC
ssODN_EMX1	TCCTCCCCATTGGCCTGCTTCGTGGCAATGCGCCACCGGTTGATGTGATGGGAGCCCTTGGATCCAAGCTTCTTCTGCTCGGACTCAGGCCCTTCCTCCTCCAGCTTCTGCCGTTTGTACTTTGTCCTC
ssODN_VEGFA	TATTGGAATCCTGGAGTGACCCCTGGCCTTCTCCCCGCTCCAACGCCCTCAACCCCACAGGATCCAAGCTTACACACTCACTCACCCACACAGACACACACGTCCTCACTCTCGAAGACGCTGCTCGCT
T7E1 H2B T Fw	GGCCCTAGAAGAGCGAGTCT

T7E1 H2B T Rv	GCGCTCGAAAATGTCGTTCACA
T7E1 AAVS1 T Fw	TTCGGGTCACCTCTCACTCC
T7E1 AAVS1 OT Fw	GCATTAGGGCTTGGTCATGT
T7E1 AAVS1 T Rv	GGCTCCATCGTAAGCAAACC
T7E1 AAVS1 OT Rv	TCCTCCCATGCTATGTCTCC
T7E1 EMX1 T Fw	GGAGCAGCTGGTCAGAGGGG
T7E1 EMX1 OT1 Fw	TCTCTCCTTCAACTCATGACCAGCT
T7E1 EMX1 OT2 Fw	TGAAATCTCACCTGGGCGAGA
T7E1 EMX1 T Rv	GGGAAGGGGGACACTGGGGA
T7E1 EMX1 OT1 Rv	ATCTGCACATGTATGTACAGGAGTCAT
T7E1 EMX1 OT2 Rv	TGCAGTCTGCCTTTTTGGGG
T7E1 VEGFA T Fw	TCCAGATGGCACATTGTCAG
T7E1 VEGFA OT1 Fw	GAGGGGGAAGTCACCGACAA
T7E1 VEGFA OT2 Fw	TCCTTTGAGGTTCATCCCCC
T7E1 VEGFA T Rv	AGGGAGCAGGAAAGTGAGGT
T7E1 VEGFA OT1 Rv	TACCCGGGCCGTCTGTTAGA
T7E1 VEGFA OT2 Rv	CCAATCCAGGATGATTCCGC
