# Supplementary material for: A cell cycle-dependent CRISPR-Cas9 activation system based on an anti-CRISPR protein shows improved genome editing accuracy
Source: Commun Biol. 2020 Oct 23;3:601. doi: 10.1038/s42003-020-01340-2 (PMC7584632; doi:10.1038/s42003-020-01340-2)
Supplement: Supplementary file 3 — Description of Additional Supplementary Files [file 42003_2020_1340_MOESM3_ESM.pdf]

Description of Additional Supplementary Files

File Name: Supplementary Data 1
Description: Source data
